# The Effect of Axon Resealing on Retrograde Neuronal Death after Spinal Cord Injury in Lamprey

**DOI:** 10.3390/brainsci8040065

**Published:** 2018-04-14

**Authors:** Guixin Zhang, William Rodemer, Taemin Lee, Jianli Hu, Michael E. Selzer

**Affiliations:** 1 Shriners Hospital Pediatric Research Center (Center for Neural Repair and Rehabilitation), Philadelphia, PA 19140, USA; kathy.zhang@temple.edu (G.Z.); rodemer@temple.edu (W.R.); tue59650@temple.edu (T.L.); jianli.hu@temple.edu (J.H.); 2 Department of Neurology, the Lewis Katz School of Medicine at Temple University, 3500 North Broad Street, Philadelphia, PA 19140, USA

**Keywords:** axon resealing, spinal cord injury, retrograde neuronal death, regeneration

## Abstract

Failure of axon regeneration in the central nervous system (CNS) of mammals is due to both extrinsic inhibitory factors and to neuron-intrinsic factors. The importance of intrinsic factors is illustrated in the sea lamprey by the 18 pairs of large, individually identified reticulospinal (RS) neurons, whose axons are located in the same spinal cord tracts but vary greatly in their ability to regenerate after spinal cord transection (TX). The neurons that are bad regenerators also undergo very delayed apoptosis, signaled early by activation of caspases. We noticed that the neurons with a low probability of axon regeneration tend to be larger than the good regenerators. We postulate that the poorly regenerating larger neurons have larger caliber axons, which reseal more slowly, allowing more prolonged entry of toxic signals (e.g., Ca^++^) into the axon at the injury site. To test this hypothesis, we used a dye-exclusion assay, applying membrane-impermeable dyes to the cut ends of spinal cords at progressively longer post-TX intervals. Axons belonging to the very small neurons (not individually identified) of the medial inferior RS nucleus resealed within 15 min post-TX. Almost 75% of axons belonging to the medium-sized identified RS neurons resealed within 3 h. At this time, only 36% of the largest axons had resealed, often taking more than 24 h to exclude the dye. There was an inverse relationship between an RS neuron’s size and the probability that its axon would regenerate (r = −0.92) and that the neuron would undergo delayed apoptosis, as indicated by staining with a fluorescently labeled inhibitor of caspases (FLICA; r = 0.73). The artificial acceleration of resealing with polyethylene glycol (PEG) reduced retrograde neuronal apoptosis by 69.5% at 2 weeks after spinal cord injury (SCI), suggesting that axon resealing is a critical determinant of cell survival. Ca^++^-free Ringer’s solution with EGTA prolonged the sealing time and increased apoptotic signaling, suggesting that factors other than Ca^++^ diffusion into the injured tip contribute to retrograde death signaling. A longer distance of the lesion from the cell body reduced apoptotic signaling independent of the axon sealing time.

## 1. Introduction

Spinal cord injury (SCI) causes permanent loss of motor and sensory functions in mammals because axons in the central nervous system (CNS) fail to regenerate. In contrast, the regeneration of descending reticulospinal (RS) axons in the sea lamprey, *Petromyzon marinus*, accompanies a nearly complete functional recovery within 5–6 weeks following complete spinal cord transection (TX) [1,2,3,4,5,6,7,8]. Despite the behavioral recovery, fewer than half of the axons regenerate and, those that do, reconstitute only part of their original length. It has been estimated that by 10 weeks post SCI, the RS axons regenerated only 1–2% of the normal numbers of synapses distal to the TX [9]. Those neurons that fail to regenerate often undergo a very delayed (16 weeks post-TX) form of apoptosis [10], but caspase activation can be detected in some of them by 2 weeks [11]. These appeared to be the largest RS neurons. Thus, we now postulate that the size of the neuron correlates with the caliber of its axon and that larger axons seal more slowly than small ones, allowing more time for toxic factor(s) to enter the axon and activate an apoptotic response. We do not attempt to identify the toxic factors. In vitro studies have suggested that Ca^++^ entry into the cut axon tip is involved in neuron apoptosis, but only if the axotomy occurred within 50 µm from the cell body [12], whereas, in the lamprey, apoptosis can occur in RS neurons axotomized more than 1 cm from the perikaryon. The lamprey RS system is a favorable preparation in which to examine the interactive roles of neuron size, axon sealing, and distance of axotomy from the perikaryon in vivo because the lamprey contains several individually identified RS neurons of different sizes, whose regenerative abilities have been mapped [2,13]. Moreover, because the regeneration is incomplete, there is ample room for experimental manipulations to either enhance or retard regeneration and cell survival.

The lamprey brainstem contains about 3000 spinal-projecting neurons, mostly RS neurons [14]. Included, are 18 pairs of individually identifiable neurons [15,16], which vary greatly in their regenerative ability [13,16,17]. The probability of axon regeneration after TX has been calculated for each identifiable neuron, indicating a range of 4–70% [13]. Because these neurons share a common extracellular environment, variations in their regenerative potential reflect neuron-intrinsic factors that determine whether an individual lamprey neuron will regenerate its axon or undergo apoptosis after spinal cord TX. In the lamprey, the poorly regenerating RS neurons selectively upregulate the expression of several genes, including UNC-5 and neogenin, which are the chemorepulsive receptors for the guidance molecules netrin and repulsive guidance molecule (RGM), respectively [18,19], protein tyrosine phosphatase sigma (PTPσ) and leukocyte antigen related phosphatase (LAR), which are receptors for the axon growth-inhibiting matrix molecules chondroitin sulfate proteoglycans (CSPGs) [11], and the synaptic vesicle-associated protein synuclein [20], which is known to accumulate in Parkinson’s and other neurodegenerative diseases. More recently, RNAseq data indicated that several highly conserved signaling pathways are upregulated after SCI in lamprey, and pharmacological inhibition of one of these, namely, Wnt signaling, inhibited axon regeneration [21]. Whether this contributes to heterogeneity in axon regeneration is not known.

Several studies have suggested that small neurons survive injury and regenerate axons better than large neurons (summarized in [22]). In these studies, it is not clear whether the small caliber of the surviving or regenerating axons reflected selective loss of large axons, atrophy of axons, or sprouting of small daughter branches from larger parent axons. These ambiguities are avoided in the lamprey because observations can be made on individually identified neurons with well-characterized spinal projections.

The delay in resealing of optic nerve axons compared to axons in the peripheral nervous system (PNS) has been postulated as an explanation for the poor regenerative ability of axons in the CNS [23]. Large myelinated axons in rat spinal roots resealed more slowly than small ones, in a process that was Ca^++^-dependent [24], but Ca^++^ levels were increased only within short distances from the point of axotomy and were restored to low levels within one day. How this affects neuronal survival is not clear. The responses of retinal ganglion cells (RGCs) to optic nerve crush would appear to contradict the notion of selective large neuron vulnerability, since the largest of these neurons, i.e., the αRGCs, survive the injury and, in mice with deletion of the phosphatase and tensin homolog (PTEN), are capable of mounting a regenerative response, whereas most smaller RGCs die [25]. This suggests that additional factors may be important in determining the vulnerability of a neuron to retrograde death.

The distance between the location of axotomy and the neuronal cell body influences gene expression and the ability of related axonal regeneration [26,27]. However, the roles of axon caliber and resealing efficiency in this phenomenon have not been investigated. In lampreys, we previously showed that retrograde neuronal death declined with the distance of the axotomy from the neuronal perikaryon [10], and this might be consistent with the attenuation of a toxic signal entering the injured axon tip. However, RS axon diameter decreases in the caudal spinal cord, so the salutary effect of distance might just reflect accelerated axon sealing due to tapering of the axon diameter. To control for this effect and to gain more insight into the roles of axon caliber, resealing time, and distance from the perikaryon in the cellular response to axotomy, we included lesions far rostrally, close to the initial segments of RS axons, where axon diameter is comparable to that of axons in the tail. We also used polyethylene glycol (PEG) to enhance axolemmal resealing following TX [28] and a Ca^++^ chelator to retard sealing. Our results suggest that axon resealing time accounts for most of the difference in the regenerative ability between neurons that regenerate well and those that regenerate poorly, but that the distance of axotomy from the perikaryon exerts an effect independent of axon sealing.

## 2. Materials and Methods

### 2.1. Animals

Larval lampreys (*Petromyzon marinus*) ranging in length from 9 to 12 cm (3–4 years old) were obtained from streams of Lake Michigan and maintained in freshwater tanks at 16 °C until the day of use. All animal procedures were performed with the approval of the Temple University Institutional Animal Care and Use Committee (ACUP#: 4610).

### 2.2. Preparation of Nissl-Stained Brain Wholemounts and Calculation of Neuronal Sizes

Eleven lampreys were deeply anesthetized by immersion in saturated aqueous benzocaine until motionless to tail pinch. Lamprey brains were dissected and flat-mounted as previously described [29]. The specimens were fixed in 10% formalin in Ringer’s solutions for 15 min, rinsed twice in Ringer’s solution for 5 min each, and stained in 1% toluidine blue solution as described by Altman and Bell [30]: 1 g toluidine blue O (Fisher Scientific), 6 g borax, 1 g boric acid, 100 mL distilled water, pH 7.6. The solution was preheated to 40 °C, and the brains were incubated for 20 min at 40 °C, then differentiated, and fixed in several washes of Bodian’s fixative (5 mL formalin, 5 mL glacial acetic acid, 90 mL of 80% ethanol) until the neurons were clearly distinguished from the background (about 7 to 8 min). The tissue was further dehydrated in 80%, 90%, 95%, and two changes of 100% ethanol, 3 min each, then cleared in cedarwood oil for 20 min and mounted onto glass slides in Permount. Brain images were captured using bright field microscopy (Nikon 80i), and the cross-sectional area of each identified RS neuron was measured digitally from the outlined profiles at the focal plane of its maximal size, using the Nikon Elements software (v 2.34, Nikon). For each identified RS neuron pair in 11 brains, a mean cross-sectional area was calculated (for each identified RS neuron type, *n* = 2 neurons per brain × 11 brains = 22 neurons). A Pearson correlation was calculated between the RS neuron cross-sectional areas and their previously determined regeneration probabilities [13].

### 2.3. Spinal Cord Transection and Dye Exclusion Assay to Determine Axon Sealing Kinetics

Lampreys were anesthetized, and the spinal cords were exposed through a dorsal midline incision and transected with iridectomy scissors at the level of the 5th gill, as previously described [29]. Axolemmal resealing was assessed by a dye-exclusion assay, using 0.5 μL of 10 MW dextran-Alexa Fluor 488 (5% in 0.1M Tris buffer, pH 7.4) applied directly to the proximal spinal cord stump immediately after TX. At 1, 2, or 3 h post-TX, the injury site was washed with Ringer’s solution, and 0.5 μL of a second dye, dextran tetramethylrhodamine (DTMR, 10 kDa, 5% in 0.1 M Tris buffer, pH 7.4), was applied to label unsealed axons. Additionally, a DTMR-soaked Gelfoam pledget was gently placed over the transected spinal cord, while the animals recovered on ice for an additional 2 h before being returned to fresh water tanks. Lampreys were re-anesthetized 1 week later, and their brains were dissected, flat-mounted, fixed in 4% paraformaldehyde (PFA) for 2 h, washed thoroughly in phosphate buffered saline (PBS), and imaged by widefield fluorescence microscopy.

The analysis was restricted to the 18 pairs of individually identified RS neurons. An axon was deemed sealed if its identifiable RS neuron was labeled by the dye applied immediately after TX (t = 0), but not labeled by a second dye applied at 1, 2, or 3 h post-TX (number of lampreys *n* = 2, 2, and 3, respectively). For each identified RS neuron type, the percentage of neurons whose axons had sealed was calculated as: 100 × total number of neurons with sealed axons/(2 neurons per animal × *n* animals). The cells were grouped into two categories, i.e., large (≥2000 µm^2^) and small–medium (<2000 µm^2^). Curves were generated using the Graphpad Prism non-linear regression tool (v 7.03, GraphPad, La Jolla, CA, USA). The % axons sealed was compared between the large and small–medium groups at each time point.

To confirm that the dextran-complexed dye was excluded from intact axons and assess axon sealing at alternate time points, additional TX and hemi-TX experiments were performed, in which only one side of the spinal cord was cut, stopping at the central canal. These studies included a DTMR application after 15, 30, 60, or 180 min post-TX, and a DTMR or D-AF488 application at 24 or 48 h post-TX. In both cases, the tissues were harvested 1 week after injury and imaged as described above.

### 2.4. Detecting Apoptosis Signaling with Fluorescently-Labeled Inhibitor of Caspases (FLICA)

To examine apoptosis signaling in neurons with axons undergoing very delayed resealing, DTMR was applied 24 h after the initial TX, and the lampreys were allowed to recover for 2 (*n* = 8), 4 (*n* = 4), and 10 (*n* = 3) weeks after injury. Afterwards, freshly dissected brains were processed using the Image-iT™ LIVE Green Poly Caspases Detection Kit (I35104, Invitrogen, Carlsbad, CA, USA), which uses the FLICA reagent, FAD-VAM-FMK, to label activated caspases. As previously reported, apoptotic lamprey RS neurons were labeled as early as 1 week after TX (peaking at 4 weeks), while these neurons did not become positive for terminal deoxynucleotidyl transferase-mediated dUTP nick-end-labeling (TUNEL) until 4 weeks after injury (peaking at 12–16 weeks) [31]. After processing, the brains were fixed in PFA, washed thoroughly with PBS, and imaged by fluorescence microscopy.

In the analysis of these assays, RS neurons in each recovery group were binned according to the combinations of dye they contained: i.e., DTMR only (not sealed, not apoptotic), FLICA only (sealed, apoptotic), and DTMR + FLICA (not sealed, apoptotic). The percent labeling after TX was calculated as: (number of labeled neurons in a bin divided by the total number of cells in the group) × 100.

### 2.5. Measuring the Effect of the Distance of TX from the Cell Body on Retrograde Neuronal Death

To determine the effect of the distance of TX from the perikaryon on retrograde neuronal death, lamprey spinal cords were transected at 20% (5th gill), 50%, or 75% body length (BL; *n* = 4 animals at each level). The animals were allowed to recover for 24 weeks, after which the brains were dissected and stained with toluidine blue to label the surviving neurons. For each identifiable RS neuron type, the percentage of cells undergoing retrograde neuronal death was calculated as: (total number of absent cells/total number of cells) × 100, where the total number of cells = 2 neurons per animal × 4 animals = 8. An overall percentage of cells that disappeared in Nissl-stained brain wholemounts was determined for each TX level as 100 × (total number of absent cells/total number of cells), where the total number of cells = 36 identifiable neurons per animal × 4 animals = 144).

### 2.6. Distinguishing Distance-Dependence of Retrograde Apoptotic Signaling from the Effects of Variation of Axon Caliber along the Length of the Lamprey’s Body

As RS axons extend into the tail of the animal, they become narrower. This was documented by measuring the cross-sectional area of the 20 largest axons in the ventromedial axon tracts, plus the two Mauthner (Mth) axons in the lateral axon tracts, at 10% (level of the 2nd gill), 20% (level of the 5th gill), 50%, and 75% (level of the cloaca) BL from the rostral end, in the spinal cord of a non-injured 12 cm lamprey. Five-mm-long samples of spinal cord encompassing each of the above levels were fixed in 4% PFA containing 1% glutaraldehyde (to reduce tissue shrinkage) in PBS for 3 h. The samples were processed, sectioned, and stained for neurofilaments (NFs) as describe below. Cross-sectional areas were converted to diameters by: D = 2 × SQRT (area/3.14).

To investigate whether the dependence of retrograde apoptosis on the distance of axotomy from the perikaryon is due simply to the distal attenuation of axon caliber, lampreys had a spinal cord TX at 10%, 20%, or 75% body length, followed by administration of the exclusion dye DTMR, 24 h after injury. The brains and spinal cords were dissected 2 weeks post-TX and fixed in PFA. The spinal cord specimens were dehydrated and embedded in paraffin. The brains were processed for whole-mounting. In preliminary experiments, we confirmed that except I6, all of the identified RS axons reach 75% BL, by applying D-AF488 to the cut end of the spinal cord immediately after TX.

Delayed axolemma resealing was assessed by widefield fluorescence microscopy of transverse sections taken from the spinal cord 1.5–2 mm rostral to the TX. Afterwards, colorimetric neurofilament staining (see below) was performed to label all of the RS axons, including those that had sealed and thus excluded the fluorescent dye. An additional cohort of lampreys were allowed to recover for 2 weeks, after which their brains were dissected and processed for FLICA. The number of DTMR (+) and FLICA (+) neurons was compared among the different TX-level groups.

### 2.7. Neurofilament (NF) Immunostaining

After fixation, the spinal cord pieces were dehydrated in serial ethanol dilutions overnight in a tissue processor and embedded in paraffin. The blocks were cut at 10 μm, dehydrated in serial ethanol dilutions, deparaffinized in two changes of toluene, and rehydrated in 5 min washes with 100%, 95%, 90%, 80%, and 70% ethanol, each performed twice. After three washes in PBS containing 0.2% tween-20, the sections were blocked with 10% FBS in wash solution for 30 min and incubated with a lamprey NF-specific monoclonal antibody (LCM 16 at 1:100 in blocking solution) overnight at 4 °C in a humidified chamber. After three washes in PBS, the primary antibody was detected by an avidin–biotin complex (ABC) immunohistochemistry kit (Vectastain; Vector Laboratories, Burlingame, CA, USA), using the manufacturer’s protocol. The sections were developed colorimetrically by diaminobenzidine (DAB) chromogen substrate for 5 to 10 min, then washed, dehydrated, cleared, and mounted in Permount.

### 2.8. Accelerating Axon Sealing with PEG

To test the effect of PEG on axon resealing and retrograde neuronal death, 40% PEG in Ringer’s solution was applied for 5 min to the spinal cord stump immediately after TX at the level of the 5th gill. Then, the area was washed with Ringer’s solution, and the animals were allowed to recover on ice for 2 h before being returned to fresh water tanks. Control lampreys received TX only. Delayed axon resealing was determined by the application of DTMR 24 h post-injury. The brains were dissected two weeks after injury and processed by the polycaspase FLICA assay. The number of DTMR+ neurons and FLICA+ neurons were compared between animals treated with or without PEG.

### 2.9. Statistical Analysis

Comparisons between continuously variable data sets were analyzed using Microsoft Excel, GraphPad InStat 3 (GraphPad, La Jolla, CA, USA), and GraphPad Prism software. Statistical analysis was performed by ANOVA and post-hoc two-tailed *t*-test. Correlation analysis was by Pearson Correlation test. All values were expressed as mean ±SEM. Non-parametric variables were analyzed by Spearman’s rank correlation.

## 3. Results

### 3.1. The Probability of Axonal Regeneration Correlated Inversely with Neuron Size

**Figure 1:**
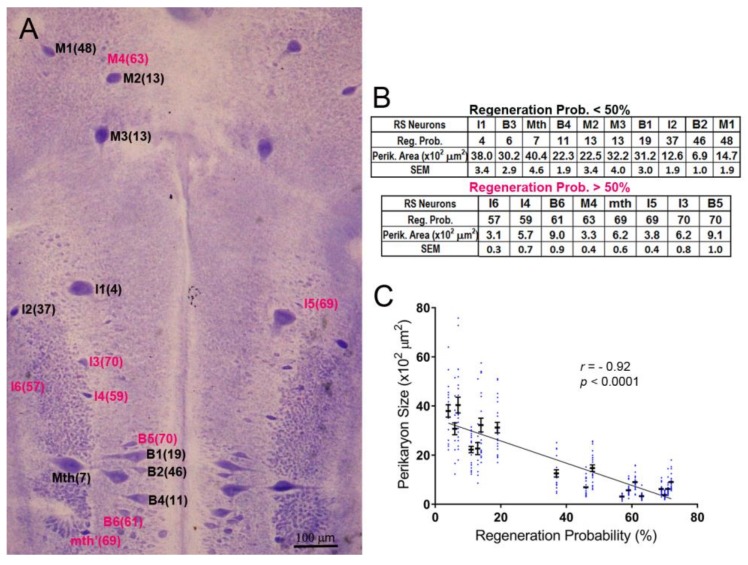
The sizes of identified reticulospinal (RS) neurons correlate inversely with their axon regeneration probabilities. (**A**) Toluidine blue-stained whole-mount of a lamprey brain. The identified RS neurons are labeled, with the % probability that their axon will regenerate by 10 weeks post-TX [13] shown in parentheses. Good regenerators are labeled in black, bad regenerators in red; (**B**) tables listing the probability of regeneration for each of 18 paired, identified RS neurons and their cross-sectional area (mean and SEM) from 11 larval lampreys; (**C**) scatter plot showing that the regenerative probabilities of RS neurons are inversely correlated with their sizes. The neuron names in **A** and **B** are according to the nomenclature of Rovainen (1976). B = bulbar group; I = isthmic group; M = mesencephalic group; Mth = Mauthner neuron; mth’ = auxiliary Mauthner neuron.

The neuronal cytoarchitecture of the lamprey brain [16] is illustrated in a whole-mount preparation stained with Toluidine Blue (Nissl stain; Figure 1A). For each identified RS neuron type, the probability of axon regeneration is shown in parentheses. These probabilities were determined previously by retrograde labeling in 27 lamprey spinal cords examined 10 weeks post-TX [13]. Neurons with regeneration probability equal to or higher than 50% were defined arbitrarily as good regenerators (deep pink color in Figure 1A,B), and the others as bad regenerators (black labels in Figure 1A,B), although their sizes and regenerative abilities varied continuously. For the most part, the bad regenerators were the largest RS neurons, except for I_2_ and B_2_, which had intermediate regeneration probabilities. The inverse relationship between the axons’ regeneration probabilities and the sizes of their cell bodies was highly significant (r = 0.92, *p* < 0.0001; Figure 1C).

### 3.2. Axon Sealing of Identified RS Neurons after Injury

**Figure 2:**
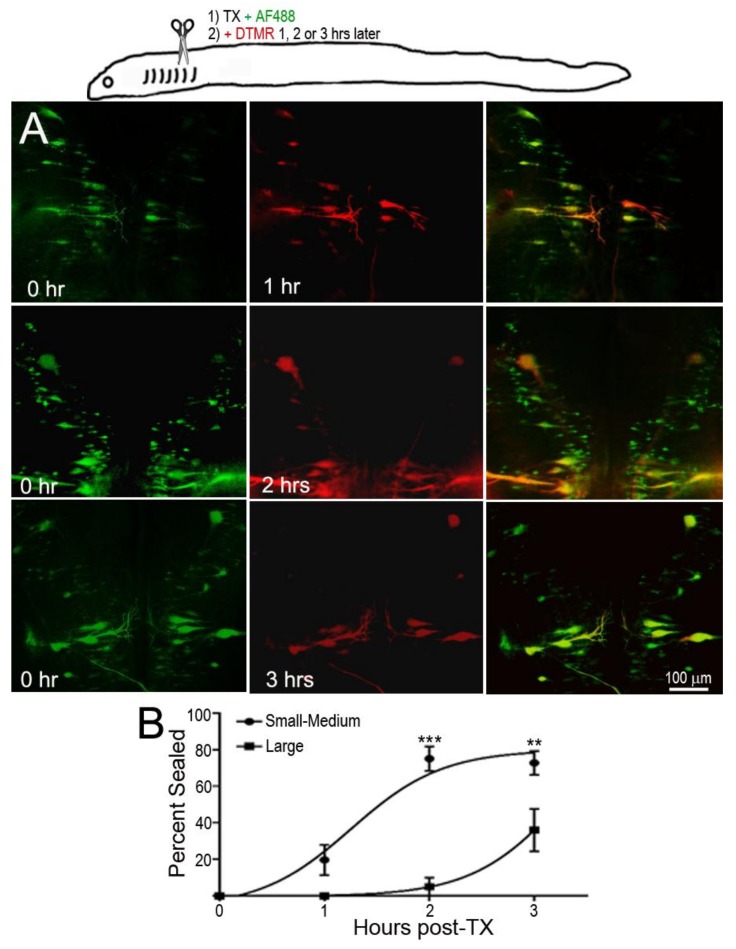
Delayed axon resealing of large neurons. RS neurons were labeled retrogradely by D-AF488 applied to fresh spinal cords transections (TX) made at the level of the 5th gill in three animals. DTMR was applied to the same site 1–3 h later, as diagrammed in the drawing at the top. (**A**) **Left column**, RS neurons in three different animals labeled by D-AF488; **middle column**, in the same three brains, RS neurons labeled by DTMR applied at 1, 2, or 3 h post-TX, as indicated; **right column**, overlay images show that the axons of small RS neurons resealed as early as at 1 h post-TX, but the axons of large RS neurons remained open until 3 h post-TX; (**B**) non-linear regression curves of % of axons sealed. “Large” neurons are identified RS neurons with cross-sectional areas ≥2000 µm^2^; the other neurons are “Small–Medium”. For the Small–Medium neurons, r = 0.85; for the Large neurons, r = 0.92. For the differences between the Small–Medium and the Large groups, ** *p* = 0.0032, *** *p* < 0.0001.

**Figure 3:**
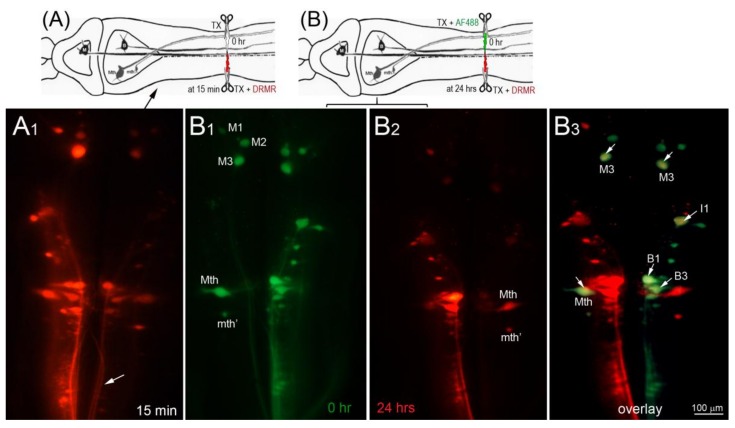
Most damaged axons of large RS neurons failed to seal completely at 24 h after spinal cord TX. In this figure, drawing (**A**) diagrams the experiment in micrograph (**A1**), and drawing (**B**) diagrams the experiments in micrographs (**B1**–**B3**). (**A**) A right hemi-TX was made at the level of the 5th gill. The TX was completed 15 min later with a left hemi-TX, and DTMR (red) was applied to the TX site. The small non-identified neurons of the left medial-inferior RS neurons are unlabeled (white arrows), indicating that their axons have sealed; (**B**) D-AF488 (green) was applied to a fresh right hemi-TX at the 5th gill (**B1**). (Note, the left M_1_, M_2_, and M_3_ neurons are unintentionally labeled because their axons are located very near the midline in the spinal cord); at 24 h post-TX (**B2**), DTMR was applied to a fresh left hemi-TX at the same level. RS neurons not labeled with D-AF488 in (**B1**) were strongly labeled with DTMR, but some large RS neurons labeled with D-AF488 were also lightly labeled with DTMR (white arrows in overlay image of (**B3**), indicating that their axons were not completely sealed by 24 h post-TX. These neurons are bad regenerators.

In the dye-exclusion assay, D-AF488 was applied to freshly TXed spinal cords at the level of the 5th gill (20% BL; Figure 2A, left column), and then DTMR was applied at the original TX site 1 h (*n* = 2), 2 h (*n* = 2), and 3 h (*n* = 3) post-TX (Figure 2A, middle column). Overlay images (Figure 2A, right column) showed that the axons of small RS neurons sealed faster than those of large neurons. At 1 h post-TX, all axons of large neurons remained open. At 2 h post-TX only 5% of large axons were sealed, and, even at 3 h post-TX, only 36% of these axons were sealed. In contrast, over 75% of axons belonging to small–medium neurons had sealed by 2 h post-TX. Thus, the axon sealing time correlated significantly with the perikaryon size (Figure 2B). Since the labeling intensity of the two dyes is different (the green D-AF488 is usually brighter than red DTMR), the results were confirmed with an internal control, i.e., a right hemi-TX was made at the level of the 5th gill, and, 15, 30, 60 min, and 3 h later, a left hemi-TX was added, and DTMR was applied to the full transection site. The axons belonging to the very small neurons (not individually identified) of the medial-inferior RS nucleus were sealed as early as 15 min post-TX (white arrow in Figure 3A), but the axons of some large identified RS neurons remained open even at 3 h post-TX (not shown). Therefore, we extended the sealing time to 24 h (Figure 3B1–B3) and 48 h post-TX. To label the axons transected originally, D-AF488 was applied to freshly hemisected spinal cords on the right side at the 5th gill (Figure 3B1). The axons of Mth and auxiliary Mauthner (mth’) neurons decussate, and, therefore, these cells were labeled contralateral to the Müller neurons. A second fluorescent dye (DTMR) was applied to a left hemi-TX at 24 h post-TX (Figure 3B2). The RS neurons whose axons were not sealed were doubly labeled (arrows in Figure 3B3). The labeling with the second dye was not as strong as the labeling with the first dye (t = 0), suggesting that these axons were partially sealed by 24 h. The neurons labeled by the second dye were all bad regenerators. In some cases, the I1 and Mth neurons, which were the worst regenerators [13], had not sealed completely by 48 h post-TX (they were very faintly labeled; not shown).

### 3.3. Most RS Neurons with Delayed Axon Sealing Were FLICA Positive

**Figure 4:**
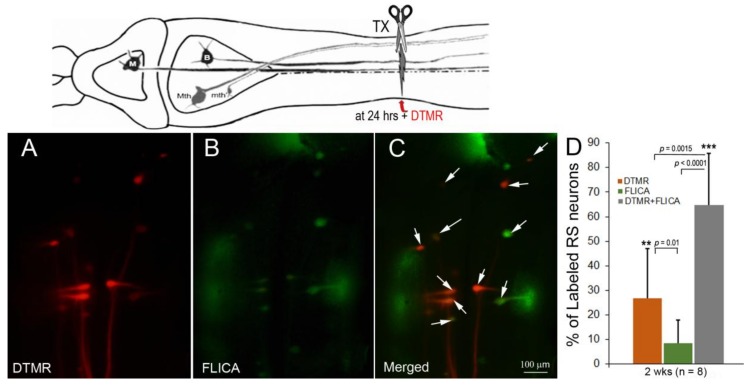
Association between delayed axon sealing and retrograde apoptosis signaling. Neurons with delayed axon sealing were retrogradely labeled with DTMR (red) applied to the spinal cord TX 24 h post-TX (**A**); after 2 weeks, the brains were processed for FLICA (**B**); the white arrows in the merged images (**C**) point to double-labeled neurons (many are faint); (**D**) counts of DTMR-labeled, Fluorescently-Labeled Inhibitor of Caspases (FLICA)-labeled, and double-labeled neurons. Most of the neurons were double-labeled. In the cartoon, M represents a mesencephalic Müller neuron; B represents a bulbar Müller neuron.

The previously determined probabilities of regeneration for individually identified RS neurons ranged between 3.7 and 70.4% at 10 weeks post-TX [13]. The numerous poorly regenerating neurons eventually die after extended survival times [10]. To determine whether delayed axon sealing was associated with activation of caspases, we combined the dye exclusion assay with FLICA labeling. The RS neurons with delayed axon sealing were labeled by application of DTMR 24 h post-TX, and FLICA staining was performed 2 weeks later (Figure 4). Most RS neurons with delayed axon sealing were also FLICA+, and most FLICA+ neurons showed delayed axon sealing. Similar results were obtained with animals at 4 (*n* = 4) and 10 (*n* = 3) weeks post-TX (not shown).

### 3.4. Distance-Dependence of Retrograde RS Neuron Death

**Figure 5:**
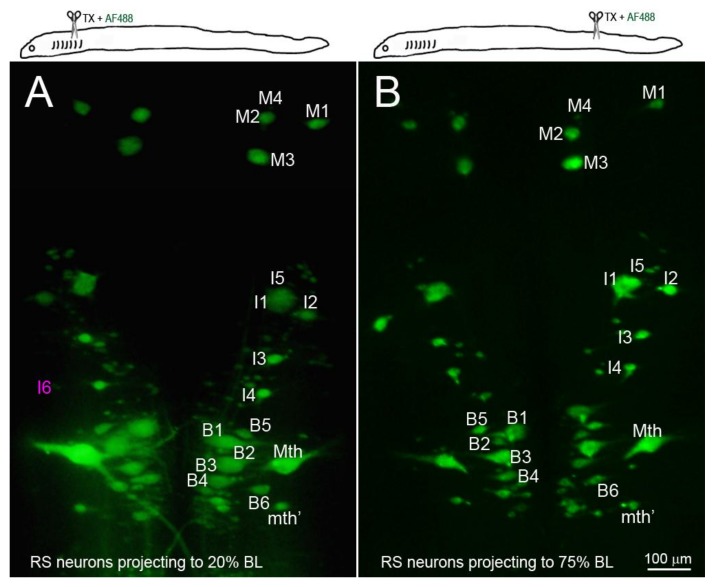
The identified RS neurons projected at least to 75% body length (BL). RS neurons were retrogradely labeled by D-AF488 applied to a fresh TX at 20% BL (5th gill, (**A**)) or 75% BL (**B**). The brains were removed and fixed 2 weeks post-TX. All the identified RS neurons except I6 (faintly seen in (**A**), magenta label) project to at least 75% BL.

**Figure 6:**
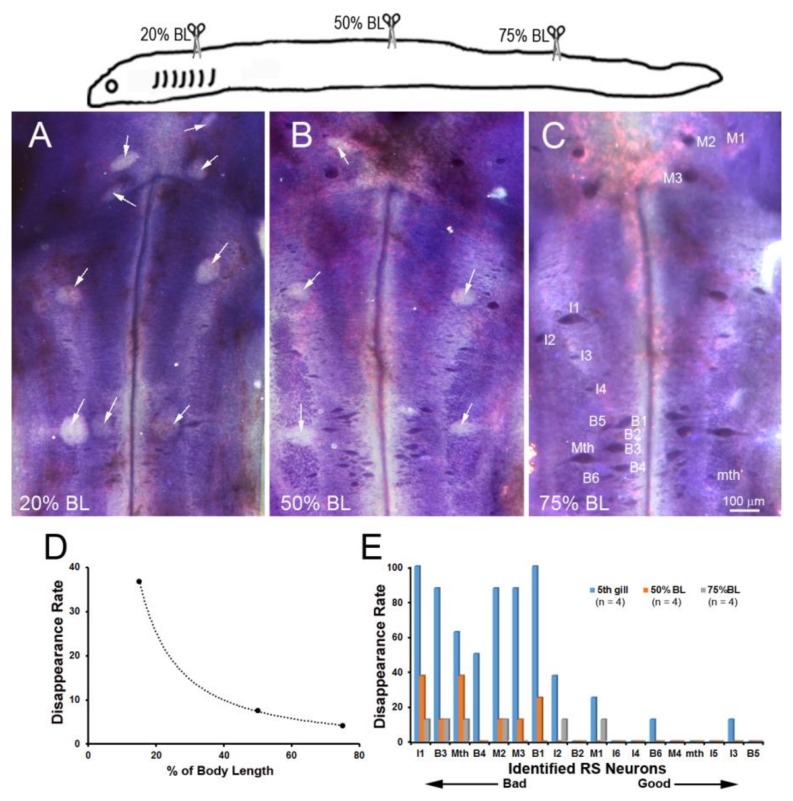
Distance-dependence of retrograde neuronal death. Neuronal death was demonstrated by disappearance of giant RS neurons in Nissl-stained brain whole-mounts 24 weeks post-TX. (**A**) Spinal cord TX at the 5th gill (20% BL) led to the disappearance of many identified RS neurons (white arrows); (**B**) TX at 50% BL, showed fewer lost RS neurons (white arrows); (**C**) most RS neurons survived when TX was at the level of the cloaca (75% BL); (**D**) the probability of death is shown for the identified RS neurons after TX at each level; (**E**) the probability of cell death decreases with the regenerative ability and distance of TX from the perikaryon.

Retrograde labeling with D-AF488 from TXs at 20% (Figure 5A) or 75% BL (Figure 5B) showed that all but one of the identified RS neurons (I6) projected at least as far as 75% BL, confirming our previous report using a chromogenic tracer [16]. In order to assess the relationship between regenerative ability and actual retrograde neuronal death (as opposed to apoptotic signaling), Nissl-stained brain whole-mounts were prepared 24 weeks post-TX, and the identified RS neurons were counted. Unstained neurons were assumed to have died or to be in a state of advanced apoptosis (arrows in Figure 6A–C). After TX at 20% BL, 36.8% of identified RS neurons had disappeared. However, when TXs were made at 50% and 75% of BL, only 7.6% and 4.2% of RS neurons were missing, respectively (Figure 6D). The probability of death for individually identified RS neurons is shown in Figure 6E, with the neurons arranged in the order of regenerative probability from bad (left) to good (right). Most of the missing neurons were bad regenerators.

### 3.5. The Sealing of Axons Depends on Their Caliber, Not on the Distance of Their Axotomy from the Perikaryon

**Figure 7:**
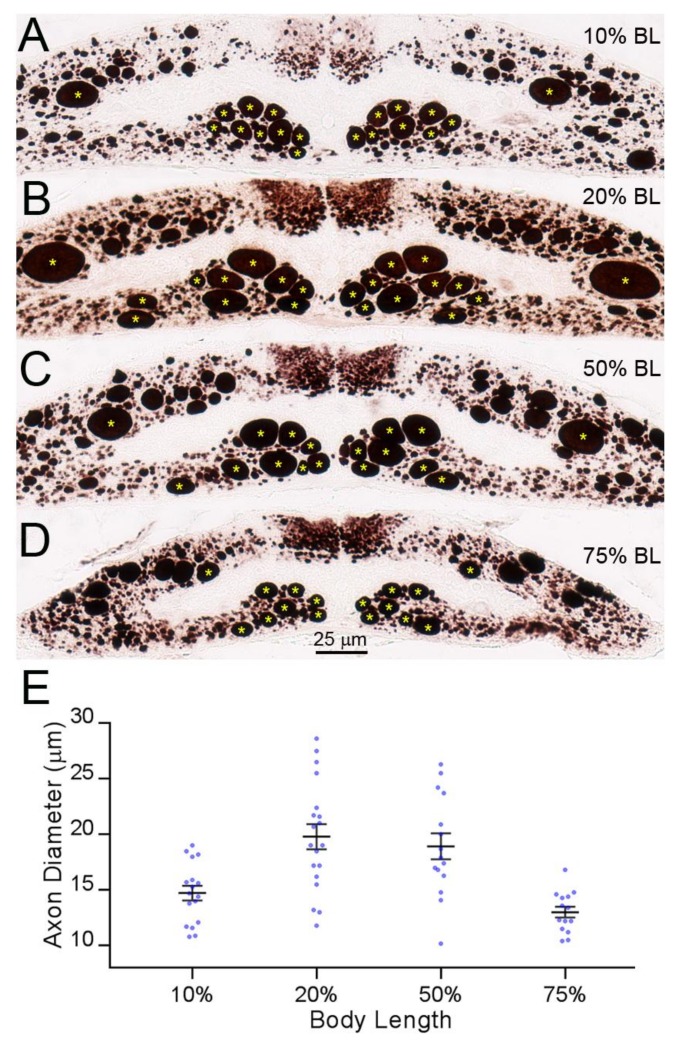
RS axon calibers vary along their course in the lamprey body. (**A**–**D**) Transverse sections along the length of the spinal cord stained with an anti-neurofilament (NF) antibody. The largest axons in the ventro-medial white matter belong to identified RS neurons (Müller cells), and the large axons in the dorsolateral column belong to the Mth neurons; (**E**) diameters of the large RS axons (*) along the body length (means ± SEM). Axon diameters at the 10% and 75% BL are comparable.

**Figure 8:**
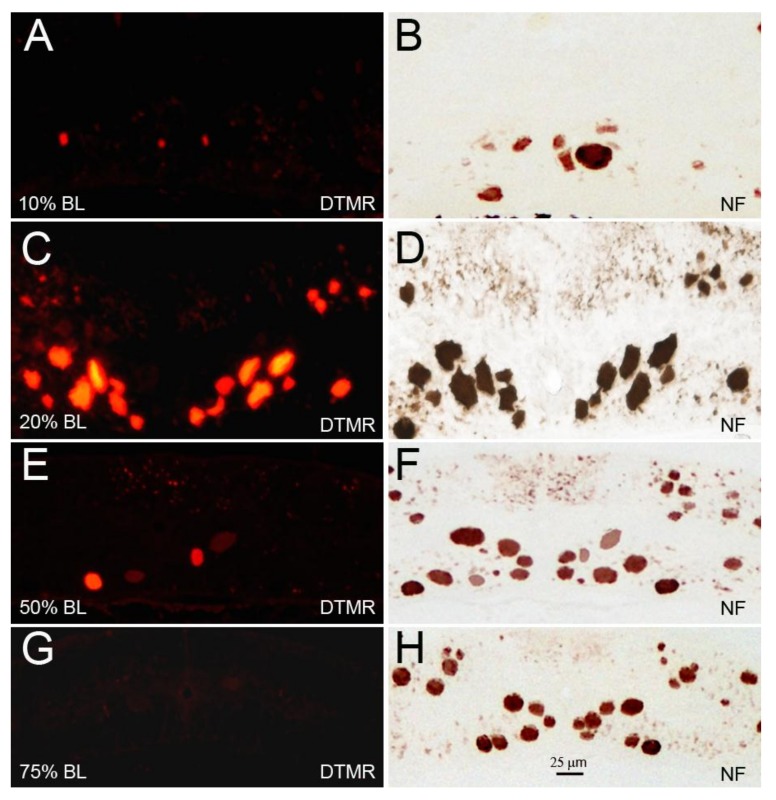
The diameter of RS axons is a determinant of axon resealing time. Spinal cords were transected at 10% (**A**,**B**), 20% (**C**,**D**), 50% (**E**,**F**), or 75% BL (**G**,**H**). DTMR was applied to the TX sites 24 h post-TX to label late-sealing axons. Transverse sections were obtained 1.5–2 mm rostral to the TX sites 2 h after dye application (**A**,**C**,**E**,**G**). Most of the RS axons had not resealed when the TX was at 20% BL, where axon diameters were maximal. When cut at 75% BL, where axons diameters were the smallest, all axons had resealed; (**B**,**D**,**F**,**H**) the same sections were stained with an anti-NF antibody to show both the sealed and the non-sealed axons.

RS axons are very narrow in the initial segment, gradually widen in the gill region, and then attenuate again in the tail. We measured the calibers of RS axons at four different distances along the BL: 10% (2nd gill), 20% (5th gill), 50%, and 75% (cloaca) BL. At 10% BL, the diameters of RS axons were smaller than they were at the level of the 5th gill and comparable to those at 75% BL [32]. This allowed us to compare the sealing of the same axons at locations where they had similar calibers, but at two different distances from the perikaryon (Figure 7). The average diameters of the large RS axons of the medial-ventral white matter (the undecussated Müller axons) and of the decussated Mth axons in the dorsolateral tract at 10% BL (14.7 ± 0.7 µm) were similar to their diameters at 75% BL (13.0 ± 0.5 µm), and their diameters were the greatest between 20% BL (19.8 ± 1.2 µm) and 50% BL (18.9 ± 1.1 µm). The dye-exclusion test showed that axon sealing was related to axon caliber, not to the distance of axotomy from the perikaryon (Figure 8).

### 3.6. Axotomy Distance and Axon Sealing Time Have Independent Effects on Retrograde Neuronal Death

**Figure 9:**
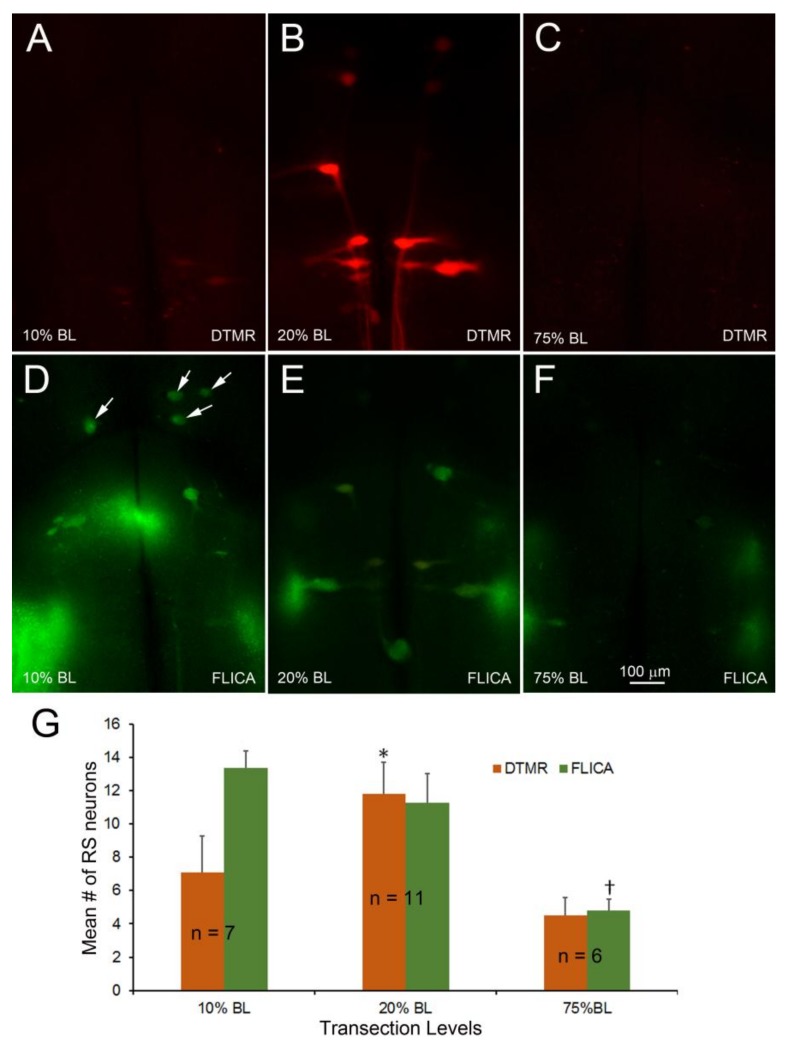
The distance from the perikaryon reduces apoptotic signaling independently of the axon sealing time. TXs were made at 10%, 20%, or 75% BL. DTMR was applied 24 h later to label late-sealing RS neurons. The animals were allowed to survive for 2 weeks, and their brains were processed for FLICA staining. (**A**–**C**) sealing was least complete when the TX was at the 20% BL, where axon caliber is the largest; (**D**–**F**) FLICA labeling was the greatest when TX was at the 10% BL—i.e., closest to the perikaryon—significantly greater than when TX was at 75% BL, even though axon resealing was not significantly different (**G**). * *p* = 0.018 for the difference in the number of DTMR-labeled neurons between 20% BL and 75% BL, † *p* = 0.018 for the difference in the number of FLICA-labeled neurons between 20% BL and 75% BL, and *p* = 0.0001 between 10% BL and 75% BL.

DTMR was applied to TXs at 10, 20, or 75% BL 24 h post-TX. Two weeks later, the brains were processed for FLICA (Figure 9). Few large RS neurons were labeled after TX at 10% BL and 75% BL (Figure 9A,C), but most were labeled after TX at 20% BL (Figure 9B), indicating that the axons transected at 20% BL had not sealed, but at 10% BL and 75% BL, they had. The proportion of RS neurons that were FLICA+ decreased with the distance of the TX from the brainstem. After TX at 10% BL, this proportion was not significantly different from that after TX at 20% BL (Figure 9D,E,G) but significantly increased compared to that after TX at 75% BL (Figure 9D,F,G). Interestingly, the identified RS neurons whose axons sealed in 24 h (no DTMR labeling) after 10% BL transection were also FLICA-positive (arrows in Figure 9D), while the same identified neurons were FLICA-negative when TX was at 75% (Figure 9F). Thus, the effect of the distance of axotomy from the perikaryon is independent of the sealing time.

### 3.7. Facilitation of Axon Sealing with PEG Reduced Retrograde Neuronal Death

**Figure 10:**
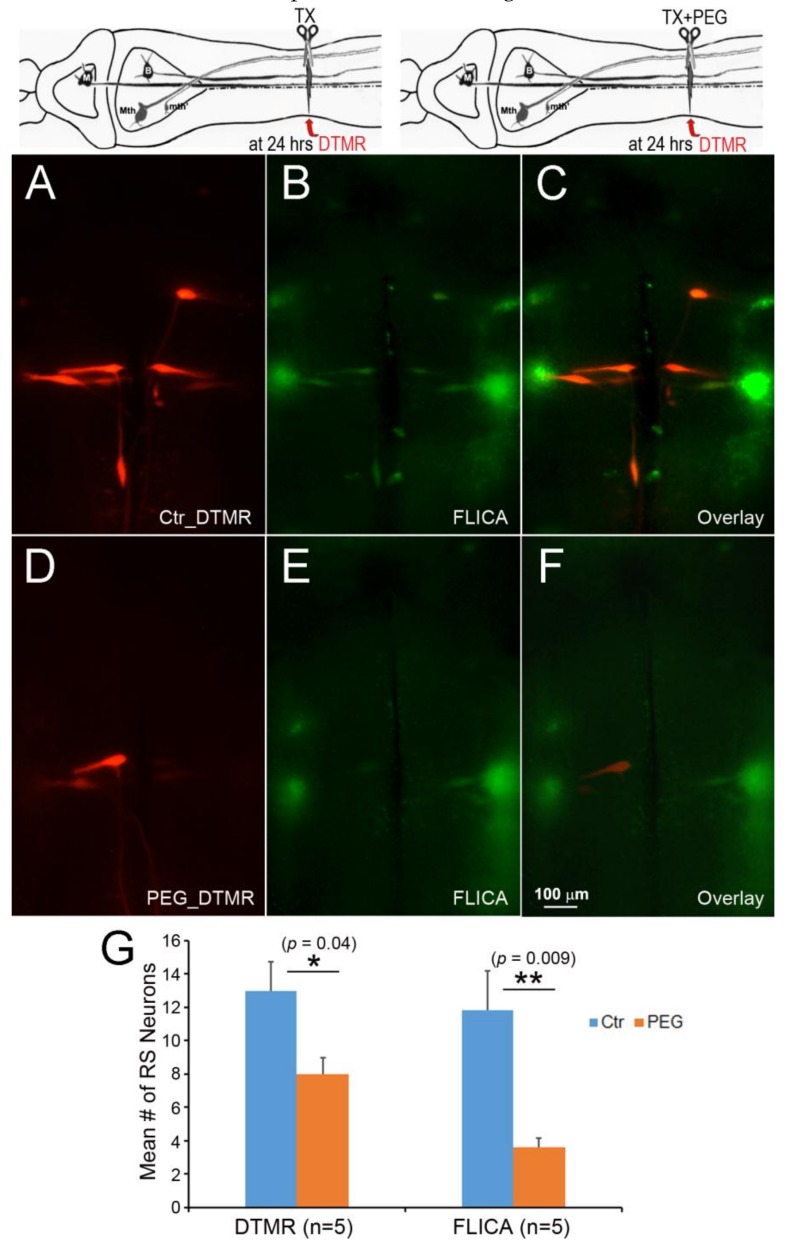
Acceleration of axon resealing with PEG reduced caspase activation. The spinal cords ere transected at 20% BL with or without PEG. At 24 h post-TX, DTMR was applied to the TX site to label neurons whose axons had not sealed (**A**,**D**); 2 weeks post-TX, the brains were processed by FLICA (**B**,**E**). PEG reduced the uptake of DTMR by severed RS axons (**A**) versus (**D**), and reduced the number of caspase + RS neurons (**B**) versus (**E**); (**G**) acceleration of axon sealing and reduction of caspase activation by PEG were both statistically significant.

To test whether delayed axon sealing is critical for retrograde neuronal death, PEG was applied to accelerate axon sealing (Figure 10). At 24 h post-TX, DTMR was applied to the TX site with or without PEG. FLICA labeling was performed 2 weeks post-TX. Compared to the control group (Figure 10A), the PEG-treated RS neurons showed greater axon resealing (Figure 10D). PEG treatment also reduced the number of FLICA+ RS neurons (Figure 10E), as compared to the control group (Figure 10B). PEG treatment facilitated axon sealing by 38.5% (*p* < 0.05) and reduced the number of apoptotic RS neurons by 69.5%, compared to controls (*p* < 0.001) (Figure 10G).

**Figure 11:**
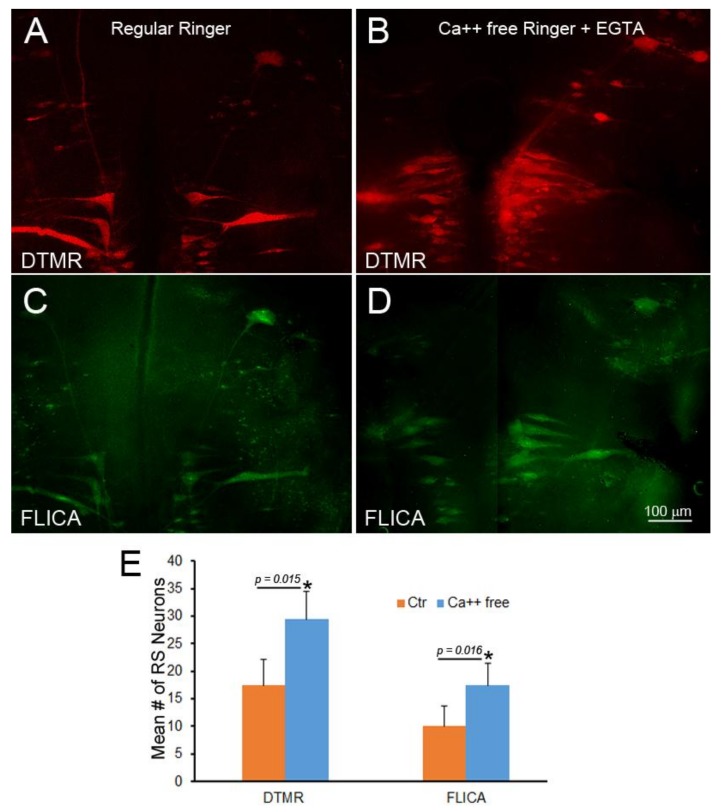
Failure of Ca^++^ chelation to protect against retrograde apoptotic signaling. Spinal cord TX was performed in Ringer’s solution containing 0 mM Ca^++^ and 1mM EGTA. After 2 h, the wound was closed, and the animals were returned to fresh water. At 24 h post-TX, DTMR was applied to the TX site to label late-sealing axons and their neurons. The animals survived 2 weeks, and their brains were processed by FLICA. (**A**) The brainstem of an animal with late-sealing axons labeled with DTMR; (**B**) similar to (**A**), but treated with normal Ringer’s solution instead of 0 mM Ca^++^/EGTA; (**C**,**D**) FLICA labeling of neurons in (**A**,**B**), respectively; (**E**) eliminating Ca^++^ increased the number of axons with delayed sealing, but increased apoptotic signaling.

To determine whether Ca^++^ entry accounted for the entire effect of delayed sealing on retrograde neuronal death, DTMR was applied to a fresh TX in seven animals, but resealing was delayed by use of Ca^++^-free Ringer’s solution with 1 mM ethylene glycol-bis(β-aminoethyl ether)-*N*,*N*,*N*′,N′-tetraacetic acid (EGTA) as the dissecting fluid, and this condition was maintained for 2 h. After 2 weeks of recovery in fresh water tanks, the brains were processed for FLICA labeling and imaged for DTMR (Figure 11). The lack of Ca^++^ at the time of TX did not protect the neurons from retrograde apoptotic signaling. In fact, apoptotic signaling was increased significantly. This suggests that factors other than Ca^++^ diffusion into the axon tip contribute to retrograde cell death.

## 4. Discussion

### 4.1. Axon Caliber is Proportional to the Size of the Perikaryon

Although, in general, large axons require large neurons to support them, obtaining quantitative support for this assumption in the CNS in vivo is difficult. Even in lamprey brain whole-mounts, with RS neurons labeled retrogradely by a fluorescent tracer, the severe attenuation of axon caliber at the initial segment often causes discontinuity in tracer visibility. However, Rovainen reconstructed the CNS from serial 4–5 μm plastic sections, tracing several spinal-projecting axons, including most belonging to individually identified RS neurons, back to their cell bodies in the brainstem in a large larva [15] and a recently-transformed young adult [32]. Although the corresponding brain sections were not shown, histological transverse sections of spinal cords were shown at several locations along the length of the cords, and the RS neurons in drawings of reconstructed brains were matched to their axons. From their published spinal cord micrographs, we measured the axons’ cross-sectional areas. We ranked the axons in order of cross-sectional area and correlated these results with the rank orders of perikaryal cross-sectional areas measured in the identified RS neurons of our own preparations. The rank orders of the axons were constant, regardless of their location along the body, and did not change between larval and young adult spinal cords. For Rovainen’s larva, whose axons were measured in the caudal gill region, the Spearman rank order coefficient r_s_ was 0.93, *p* = 0.0007. For the equivalent location in the young adult, r_s_ was 0.90, *p* = 0.0046. Even if the actual sizes were correlated, and parametric statistics was applied, the resulting Pearson correlation coefficients would be as follows: for the larva, r_p_ = 0.84, *p* = 0.0044; for the young adult, r_p_ = 0.91, *p* = 0.0016. From these findings, we could conclude that: (1) the relative sizes of identified RS neurons is stable over long periods of development, even during transformation from large larva to young adult; (2) the relative calibers of axons are constant along the length of the spinal cord and between large larva and young adult; (3) the calibers of axons are highly correlated with the sizes of their cell bodies. This allowed us to use the relative sizes of identified RS neurons as proxies for their axon calibers.

### 4.2. Axon Regeneration and Neuronal Survival after Axotomy Are Inversely Proportional to Neuron Size

In lampreys, the 18 pairs of identifiable RS neurons vary greatly in their regenerative abilities after SCI. We observed that a neuron’s size is strongly inversely correlated to its ability to regenerate its axon and to resist retrograde neuronal death. Many studies of axon regeneration after SCI, in both invertebrates and high-order mammals, have suggested that small axons and their perikarya tend to survive trauma and regenerate better than large axons. The large axons of the corticospinal tract are notoriously refractory to manipulations aimed at promoting their regeneration after SCI, whereas smaller axons, such as the serotonergic fibers of the raphe-spinal tract are much better regenerators [22]. Following bilateral crush of the cerebral-buccal connectives of *Aplysia californica*, the small axons regenerated first, gradually followed by the medium-sized axons. However, even 50 days after injury, the large-diameter axons failed to regenerate [33]. Likewise, after ischemic injury, the small axons of neurons in the dorsal funiculus of the rat spinal cord regenerated approximately 1 mm, while the large axons failed to do so [34]. A similar caliber-dependent response to injury was observed in contusion SCI in cats [35] and crush SCI in rats [36]. A source of uncertainty in all these studies is that the calibers of the regenerating neurites could be much smaller than the diameters of their injured parent axons. This makes it difficult to assess the significance of an apparent exception to this size principle, i.e., the axons of the optic nerve, which, in mice, emanate from approximately 30 different classes of RGCs [37]. The majority of these axons die after the optic nerve is crushed or cut just behind the orbit. However, the largest of them, namely, the αRGCs, selectively survive. Although, in normal animals, there is almost no axon regeneration, in mice with deleted PTEN, the axons of these αRGCs regenerate beyond the crush [25]. However, in this model, the nerve crush was made less than 1 mm behind the retina, so it is possible that RGC death was not due exclusively to axotomy, but might have included inflammatory or ischemic changes. Among the advantages of the current study in lamprey is that the neurons were individually identified, the paths taken by their axons was known, there was no ambiguity about whether their axons had been severed by the spinal cord TX, and their regenerative probabilities were well characterized. In any case, the findings about mouse αRGCs are consistent with the present study and our previous reports, in that, in the lamprey, the same neurons that are bad regenerators when tested at 7–10 weeks post-TX, show caspase activation by 2 weeks [11] and undergo delayed apoptosis by 16 weeks [10].

### 4.3. Retrograde Neuronal Death Can Be Caused by Delayed Axon Sealing

Previous studies have suggested that slow axon resealing among large neurons increases their susceptibility to cell death following injury [24,38]. Moreover, axon resealing is slower among axons of the CNS than in the PNS, which may contribute to their difference in regenerative ability [23]. Despite these findings, direct evidence linking delayed axon resealing with injury-induced neuronal death has been lacking. In the present study, because perikaryal size is a good proxy for axon caliber, it has been possible to combine retrograde labeling and FLICA to show that the RS neurons with the largest axons were also those whose axons sealed most slowly, and that apoptotic signaling was seen selectively in those slowly-sealing neurons.

### 4.4. Mechanisms of Axon Sealing

The present experiments do not address the mechanism of axon sealing, nor the nature of the toxic substances entering the axon tip that account for retrograde neuronal death and regenerative failure, except for suggesting that Ca^++^ diffusion into the injured axon tip does not account for the entire apoptotic effect. Others have explored such mechanisms. Axon sealing is Ca^++^-dependent and is blocked by phospholipase A2 inhibitors [39], and it was at first proposed that, under the influence of Ca^++^-activated phospholipase, the severed axolemmal leaflets collapsed and rapidly fused to reconstitute a continuous membrane barrier [40,41]. Such a mechanism would not easily explain the large differences in resealing times found in the present study. More recently, a substantial body of evidence has implicated a Ca^++^ influx-dependent formation of vesicles at sites of axon damage [24,38,42,43,44,45,46,47,48,49]. The vesicles migrate, accumulate, and pack tightly together at the injury site to reduce further influx of Ca^++^, until a seal is restored [42]. This is viewed as a protective mechanism because excess Ca^++^ is thought to mediate neuronal damage by activating proteases, lipases, and other target molecules [38]. Large axons would require a larger vesicle plug and thus reseal more slowly than axons requiring a smaller plug. Indeed, in transected rat dorsal roots, small-diameter axons sealed more rapidly than large ones [24]. Other laboratories have measured the intracellular Ca^++^ concentration in the proximal segments of transected lamprey axons [50]. Within 3 min after TX, spatially graded increases in Ca^++^ concentration were apparent and reached a distance of 1.6 mm from the cut end in 3 hrs. By one day post-TX, Ca^++^ concentrations dropped to pre-cut levels. Failure to reseal underlay prolonged Ca^++^ influx through micropores in the axolemma after spinal cord contusion in mouse and predicted axonal demise [51]. Since Ca^++^ influx can trigger cell death pathways but also initiates the plasmalemmal sealing that reduces Ca^++^ influx, Ca^++^ plays contradictory roles in retrograde neuronal death. This was evident from the effects of Ca^++^ chelation. When the injury site was bathed in 0 mM Ca^++^/EGTA, resealing was delayed, and caspase activation was increased. Since Ca^++^ diffusion into the axon would have been reduced, the apoptotic signaling probably was due to prolonged influx of additional toxic factors from the extracellular environment. This does not preclude the possibility that the injury activated the release of Ca^++^ from intracellular compartments; however, in cultures of B104 cells, ryanodine blockade of Ca^++^ release from internal stores did not affect cell survival [12]. SCI induces bleeding, inflammation, and macrophage activation as early as a few hours after SCI, peaking at 5–7 days. The cytotoxic products of activated phagocytes cause secondary injury [52,53,54]. Reactive gliosis also occurs around the injury site. The glial cells undergo hypertrophy and hyperplasia, eventually forming a glial scar, which releases molecules that inhibit axon growth and may also suppress neuronal survival [55,56].

### 4.5. Distance-Dependence of Neuronal Death after Axotomy

Neuronal survival decreases if axons or dendrites are transected very close to the cell body [10,12,38,57]. In vitro studies in B104 cells suggested that the Ca^++^ effect is seen only if axons are cut less than 50 μm from their perikarya, in the range of diffusion-mediated increases in Ca^++^ concentration [12]. However, in vivo, retrograde neuronal death can occur in far more distal lesions, and the experiments of the present study involved axotomy more than 1 cm from the perikarya in the brainstem. Because the identified RS neurons project the entire length of the body, they could be used to determine more precisely the distance effects. RS neurons axotomized at 75% BL resealed faster than they did when axotomy was at 20% BL (5th gill) and also experienced less retrograde cell death, consistent with previous findings, but, at 20% BL, axon diameters were maximal, so it was not clear whether the difference reflected a primary effect of the distance or of the axon diameter. However, the efficiency of sealing was comparable for the same axons at widely different locations (10% and 75% BL), where their caliber was similar. Thus, the distance-dependence of retrograde neuronal death is not explained by axonal sealing efficiency, and an independent effect of proximity to the perikaryon must be present. The precise mechanisms of this distance effect is not known, but these RS axons make synapses en passant along their entire length [58,59,60], and thus axotomy at different distances could deprive the perikaryon of varying amounts of target-derived trophic support.

### 4.6. PEG Facilitates Axon Resealing and Prevents Retrograde Neuronal Death

PEG facilitated axon resealing of lamprey RS axons. The Ca^++^-dependent accumulation of membrane-bound structures (mostly vesicles) that seal the injured axon is mediated by several protein isomers, many of which are involved in membrane fusion at synapses or the Golgi apparatus. These include synaptobrevin, syntaxin, synaptophilin, TRIM protein, PKA, and PKC [47,61]. PEG bypasses these complex endogenous sealing pathways and induces membrane fusion rapidly and directly by removing water, thus allowing membrane lipids to collapse, fuse, and seal the cut axon ends [61]. This mechanistic simplification strengthens the interpretation of the protective effect of PEG in retrograde RS neuronal death and provides further evidence that delayed axon resealing is a cause of the distinct susceptibility of large neurons to retrograde cell death. PEG did not induce resealing of all axons by 24 h post-TX, the largest caliber axons being the most resistant. Further studies will determine whether the efficiency of PEG treatment can be increased.

### 4.7. PEG Did Not Prevent Wallerian Degeneration

As in other vertebrate species after axotomy, the proximal end of a severed axon initially retracts [8,62,63] and subsequently attempts to regenerate, while the distal stump undergoes Wallerian degeneration. Previous studies by others suggested that PEG promotes axon repair and functional recovery by allowing the proximal and distal cut ends to fuse and re-establish the original axonal integrity [64,65,66]. In the current study, this was not observed. Rather, the distal axons underwent Wallerian degeneration as usual, while the proximal axon stumps retracted. We did not carry out a systematic quantitation of these processes but, in NF-stained sections, we noted reduced signs of proximal retraction. The effect of PEG on axon retraction and subsequent regeneration are the subject of ongoing investigations. 

## 5. Conclusions

**Figure 12:**
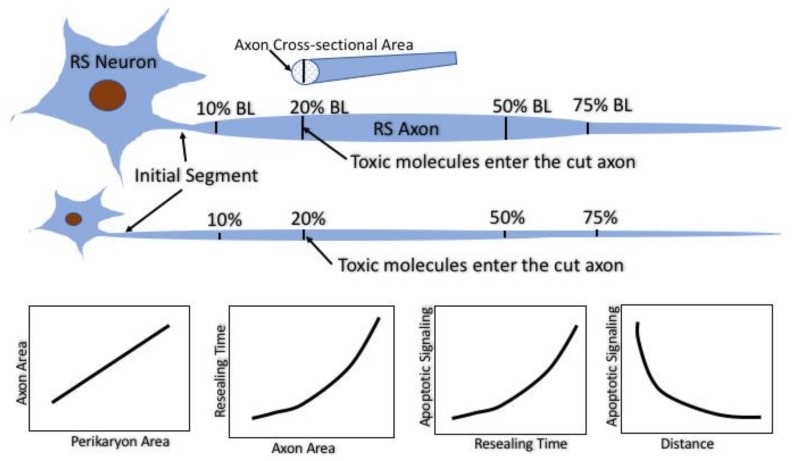
Summary of the experimental strategy and main conclusions. The identified RS neurons in the lamprey vary in size but project along almost the entire length of the animal. Their axon calibers are tightly correlated with their perikaryal size. Spinal cord TX at very rostral and very caudal levels results in axotomy where the axons are narrow and therefore seal rapidly. TX at 20% or 50% body length axotomizes the same axons at levels where their calibers are larger and they seal more slowly. At any TX level, the larger the neuron and its axon, the longer it takes for its axon to seal. The longer the sealing time, the more likely it is that the perikaryon will undergo delayed retrograde cell death. However, for any neuron, the further the TX from its perikaryon, the less likely it is that it will undergo cell death. Thus, the distance influences retrograde apoptosis independently of the sealing time.

The lamprey contains individually identified RS neurons with known projections along the spinal cord. This, together with the ability to examine these neurons and their axons in the living animal and in CNS whole-mount preparations, has been exploited in the present study to clarify ambiguities in the literature concerning the role of axon resealing in retrograde neuronal death (diagrammed in Figure 12).

Several conclusions can be drawn, at least with regard to these RS neurons. (1) The sizes of neurons are tightly correlated with the caliber of their axons; (2) The sizes of neurons are inversely correlated with their abilities to regenerate their axons after TX; (3) The sizes of RS neurons are positively correlated with their susceptibility to retrograde cell death; (4) Large caliber axons reseal more slowly than small axons, and the largest axons may remain unsealed for more than 24 h post-TX; (5) Facilitation of resealing with PEG protects against retrograde cell death; (6) The reduced susceptibility of neurons to axotomy with increasing distance of TX from the perikaryon is independent of the tapering of the axon; (7) Ca^++^ entry into the injured axon tip cannot account for the entire effect of delayed resealing on retrograde cell death. It remains for future research to provide the mechanistic details of each of these conclusions and to translate them into therapeutic opportunities for CNS injury.
